# New products

**DOI:** 10.1155/S1463924600000262

**Published:** 2000


Journal of Automated Methods & Management in Chemistry, Vol. 22, No. 5 (September–October 2000) pp. 162–164

### New products

### Dedicated FT-IR spectrometer for pharmaceutical laboratories

Within the highly regulated pharmaceutical industry, quality and precision are what you expect from your instrument supplier. This is exactly what Bio-Rad can deliver with the new PharmalyzIR FT-IR spectrometer.

The PharmalyzIR is a complete analyser dedicated to the needs of the modern pharmaceutical laboratory. Using multi-level software, the PharmalyzIR can be used for routine QC measurements of incoming raw materials, intermediates or finished products using simple single-button operations. Or at an advanced level, the software can be used for method development.

All in one package, but there is more to PharmalyzIR.

- **Security:** The multi-level software combines ease of use with a sophisticated development tool.
- **Ease of use:** Simple dialogue boxes and single-button operation for routine QC/QA.
- **Validation:** Third party software validation, verifiable training documentation, and IQ/OQ are just three of the tools we provide.
- **After sales support:** We provide a dedicated team for technical support, and several levels of service contracts.

*For more information contact: Bio-Rad Laboratories Ltd, Spectroscopy Division, Ms A. Gaches, Maylands Avenue, Hemel Hempstead, Hertfordshire HP2 7TD, UK. Fax: + 44 (0) 1442 391321*

### Fast GCMS analysis

With the ever increasing demand for faster analysis times and higher throughput of samples, we have optimized the design of our GCMS to cope with the stringent requirements of the Pharmaceutical, Environmental, Health and Contract laboratories. Shimadzu's QP5000 GCMS is simple to use and easy to maintain. It incorporates the GC-17A gas chromatograph with a Split/Splitless capillary injector, advanced automatic column flow and split ratio control.

Simple, easy to use software has been designed with automatic start up, shutdown, tuning and mass calibration capability, all available at the touch of a button. The reporting function will take the peaks found in a total ion chromatogram and automatically average the spectrum, subtract the background, search the NIST 98, Wiley or PMW Library, and provide the top 20 closest fits for each peak. The NIST library has a massive 107 000 spectra and structures, this increases the chance of finding the compound you are looking for. Fragrance, Flavour and Polymer libraries are also available. If the mass of the compound is known, then a single selective ion, monitoring analysis can be performed. This could be useful if you are looking for trace femptogram levels in environmental or medical samples.

When running fast GCMS on short microcapillary columns, it is essential to scan the peaks at a high rate so that enough data can be collected to produce accurate spectra for identification and quantitation. The QP5000 is designed for fast GCMS and has a scan speed of up to 6750 scans per second. If you scan peaks of less than 1-s width with a speed lower than 5000 scans per second, the intensity ratios change which can lead to the wrong identification and poor quantitation.

If you are running a large number of samples, it is very annoying when a filament burns out half way through the batch. In anticipation of this, a dual filament ion source with automatic switch over has been incorporated so no time is lost.

For easy cleaning, the ion source has been designed to be removed without tools and in two easy to clean parts. Plug-in filaments can be changed without removing the ion source or the column. The PFTBA standard vial is conveniently positioned below the door to the ion source, allowing level monitoring and filling up as a matter of routine.

The ceramic barings of the oil-free turbomolecular pump are corrosive resistant, the design has been guaranteed to last 25 000 h (twice as long as similar pumps). This long-term reliability and rugged design means the instrument up time is improved and service costs are minimized, this allows us to offer a low-cost, fully comprehensive service contract.

In conjunction with the AOC-20s autoinjector, the QP5000 can run up to 150 samples overnight or during the morning if fast GC capability is used. The autoinjector is mounted on pre-aligned posts, it can be lifted off the injection port to replace the septa and then simply drop back into place with no realignment needed. The syringe has also been designed to easily lift out of the cradle that supports it and has been fitted with a needle guide to ensure its position.

A range of free application notes on GCMS are available from Shimadzu UK.

*For more information please contact: Jane North, Shimadzu UK, Mill Court, Featherstone Road, Milton Keynes, MK12 5RE, UK. Tel: + 44 (0) 1908 552200; Fax + 44 (0) 1908 552211; URL—http: //www.shimadzu.com*

### IRB 140 Robot – Small in size, big on ambition

Consumer goods manufacturers represent just one of the industries that will benefit from ABB Flexible Automation's new mini marvel – the IRB 140.

Weighing in at just 98 kg and just 81 cm high, the IRB 140 packs a real punch with a 5 kg load capacity (unique to a robot of this size), six servo axes and large-robot performance.

In a retracted position, the IRB 140 has a maximum height of 810 mm from the base. When fully extended, however, it has a reach of 1240 mm vertically and

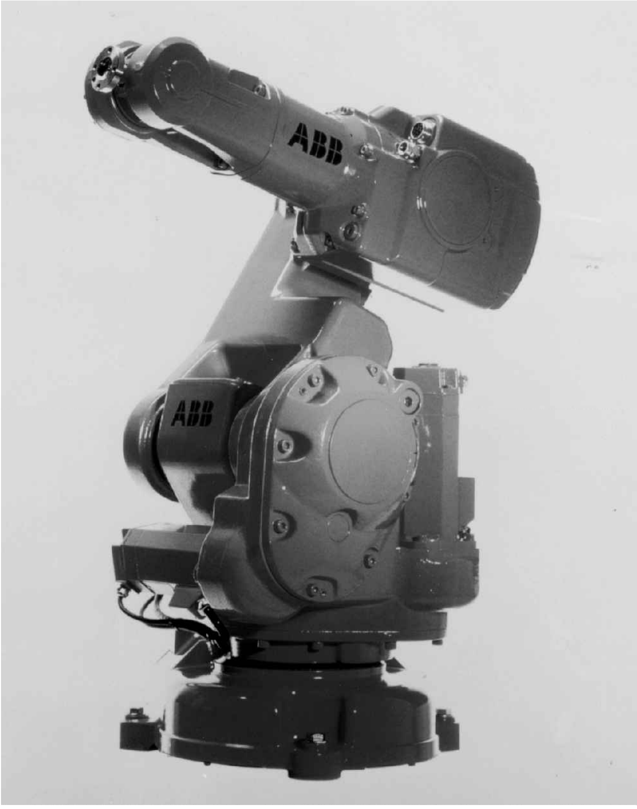

*IRB 140 robot from ABB Flexible Automation.*

810 mm horizontally, with 670 mm to the rear, all measurements to the tool interface plate on the wrist.

With a footprint of only 400 × 450 mm, the IRB 140 can be placed in an optimum position for operating – either on top of the machine, by the side of it, or even on a suspended gantry.

One of the benefits to the consumer goods industry is that the IRB 140 can be used to fill the gap between machines used for packing individual items and placing them into trays (for example confectionery) and those used for packing trays into boxes.

The new S4Cplus system – which is based on a 200 MHz Intel Pentium processor with a larger 64 Mb or 128 Mb memory size – controls the IRB 140 and offers an improved performance with wider communications capability.

A man–machine interface comes in the shape of the operator's panel which can be either cabinet or external mounted, and there is a portable teach pendant with joystick and keypad plus a display of 16 lines with 40 characters.

Higher processing speeds coupled with the large memory means faster access to installed software and rapid-start after any re-boot. Utilizing the S4Cplus means there is a high degree of PC compatibility leading to easy program file management and use of PC-type functions to aid programming.

The IRB140 is available in several variants – arc welding, foundry, clean room – which coupled with its outstanding performance makes it suitable for use in a wide range of manufacturing processes.

A major technical triumph for ABB, the IRB 140 is an advanced automation solution aimed at giving customers maximum advantage in the market place.

*For further information contact: Jane Attwood, ABB Flexible Automation Limited, Auriga House, Precedent Drive, Rooksley, Milton Keynes MK13 8PQ, UK. Tel: + 44 (0) 1908 350300; Fax: + 44 (0) 1908 350301; e-mail: jane.attwood@gb.abb.com.*

### Genevac launches unique Open Access Evaporation System for LC-MS installations at Pittcon 2000

Genevac, the world's leading manfacturer of centrifugal evaporation systems for pharmaceutical and R&D applications, launched a unique new Open Access Centrifugal Evaporator at the Pittsberg Conference 2000 in New Orleans, LA.

Designed as an integral part of an 'open access' LC-MS purification system for medicinal chemistry, the Genervac HT-4OA open access evaporator allows purified fractions to be loaded and unloaded without batching or balancing loads opposite each other in the sample rotor. As a result, the whole system runs more efficiently because chemists can process their purified fractions without delay caused by waiting for a full batch of samples required for balancing in a standard centrifugal evaporation system.

At any time, additional samples can be loaded into available spaces in the sample rotor or, if the rotor is full, replace dry samples that area ready for removal. The system incorporates a temperature control system and pressure control to ensure a safe sample temperature limit and rapid evaporation of water. At every stage in the process, the fractions can be easily identified.

At the Conference, the Genevac Evaporator was demonstrated as part of a new integrated LC-MS System featuring a Gilson preparative HPLC system and Finnigan AQA™ MS detector.

Genevac also demonstrated other examples of its latest technology at Pittcon.

- Mega Systems for ultra-high-throughput evaporation.

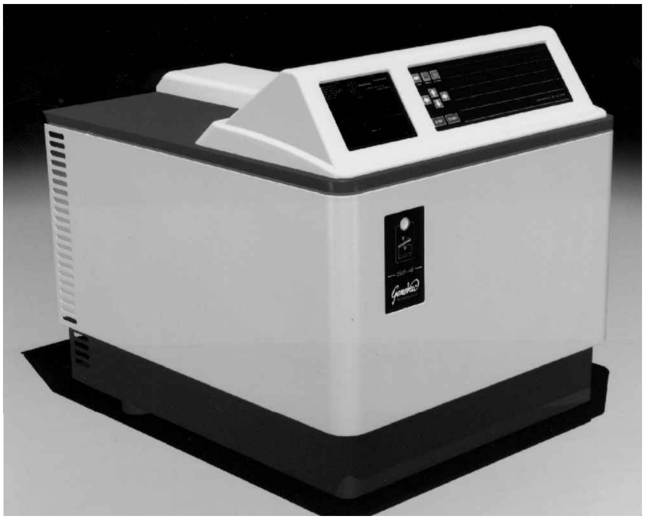

*The new Open Access Centrifugal Evaporator from Genevac.*

- HT-4 Ultra Compact Systems for medicinal chemistry.
- Parallel Synthesis Workstation.

*For more information about Genevac Open Access Evaporation Systems and the rest of the range, please contact: Harry Cole, Sales & Marketing Director, Genevac Ltd, Farthing Road, Sproughton, Ipswich IP1 5AP, UK. Tel: + 44 (0) 1473 240000; Fax + 44 (0) 1376 461176; e-mail: harry.cole@genevac.co.uk; URL—http://www.genevac.co.uk.*

### For innovation in microplate washing the clear choice is Bio-Tek Instruments

Bio-Tek Instruments recently introduced a new line of high-quality microplate washers. The MicroTek AW Series of Automated Microplate Washers is the newest addition to the innovative Bio-Tek line of analytical instrumentation. MicroTek AW washers are designed to meet 96-well washing needs and to meet the requirements of the rapidly expanding field of high-throughput screening. They provide the solution to a most important step in ELISA, FELISA, cell-based and other microplate washing applications.

The MicroTek AW Series offers high-performance fluid dispensing and ingenious mechanical design in three models. For standard 96-well microplate washing, MicroTek AW features computer control via an RS232 port, on-board programmable software for all magnetic and standard assays, and aspiration and dispense height adjustment. The MicroTek AWS was designed for 384-well and 96-well washing, and features independent adjustment of the dispense and aspirate portions of the manifold for optimal aspiration positioning, overfill washing and overflow protection, and robot compatibility. The MicroTek AWN, for processing magnetic bead assays, washes standard 96-well assays and magnetic particle assays.

As with all Bio-Tek equipment, safety and convenience were a primary concern in the design of the MicroTek AW Series. A unique priming trough eliminates the 'dummy plate' for priming and maintenance. The electronics are completed isolated from the fluidic systems.

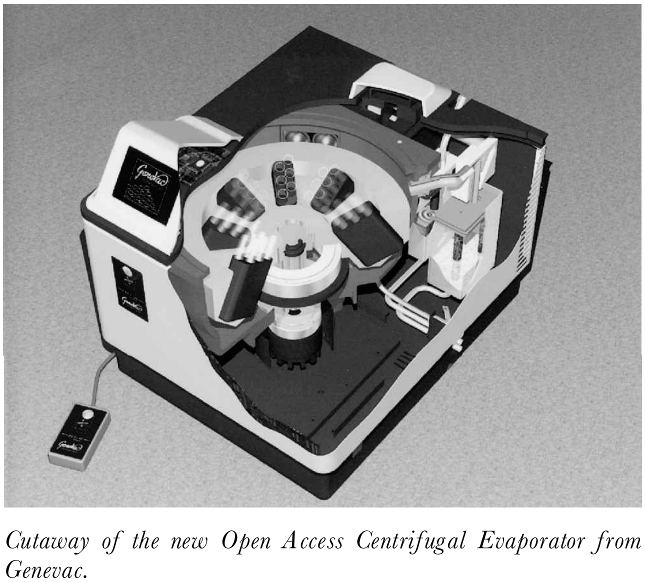

*Cutaway of the new Open Access Centrifugal Evaporator from Genevac.*

Integrated fluid and flow detection sensors minimize any potential for uneven washing. The MicroTek AW range offers the solution to ultimate precision in microplate washing.

*Issued by Tony Tingle, Wiltshire Associates, Tel: + 44 (0) 1285 640485; Fax: + 44 (0) 1285 640497. For further information please contact: Colin Fallowfield, Tel: + 44 (0) 1923 691300.*

### High speed concentration for 96-well microplates

Zymark Corporation has added to its proven line of evaporation workstations with the TurboVap® 96, a high-speed concentrator designed to work with *two* 96-well microplates or deep-well plates. With the bonus of unattended operation, the TurboVap 96 is driven by Zymark's patented gas vortex shearing action with a temperature-controlled environment and adjustable gas flow rates. The combination saves time, bench space and operating costs, while improving the evaporation speed and sample-to-sample consistency. In addition, the TurboVap 96 employs a touch pad control, which retains the previous settings for faster start-ups and has removable gas manifolds for easy cleaning.

The TurboVap 96 is the latest addition to a family of proven Zymark evaporation workstations. More than 6000 TurboVaps are in use around the world for applications such as genomics research, bioanalysis, synthesis, drug testing, drug development and environmental testing.

Zymark impacts the world by advancing the ability to discover, develop and market safe and effective drugs that can lengthen and improve the quality of life for everyone. Zymark's employees are active in more than 35 countries and their combined expertise in Pharmaceutical Analysis and Laboratory Automation makes Zymark the world's leading supplier in this application area.

*For additional information contact: Lynda Thomas, Marketing Communications, Tel: + 1 (508) 497 6549; e-mail: lnyda.thomas@zymark.com*

*The TurboVap® 96.*

